# A systematic approach to alkaline-surfactant-foam flooding of heavy oil: microfluidic assessment with a novel phase-behavior viscosity map

**DOI:** 10.1038/s41598-020-69511-z

**Published:** 2020-07-31

**Authors:** Eric Vavra, Maura Puerto, Sibani L. Biswal, George J. Hirasaki

**Affiliations:** Department of Chemical and Biomolecular Engineering, Rice University, 6100 Main St., MS-362, Houston, TX 77005 USA

**Keywords:** Energy science and technology, Fossil fuels

## Abstract

The apparent viscosity of viscous heavy oil emulsions in water can be less than that of the bulk oil. Microfluidic flooding experiments were conducted to evaluate how alkali-surfactant-foam enhanced oil recovery (ASF EOR) of heavy oil is affected by emulsion formation. A novel phase-behavior viscosity map—a plot of added salinity vs. soap fraction combining phase behavior and bulk apparent viscosity information—is proposed as a rapid and convenient method for identifying suitable injection compositions. The characteristic soap fraction, ${X}_{soap}^{Sor}$, is shown to be an effective benchmark for relating information from the phase-viscosity map to expected ASF flood test performance in micromodels. Characteristically more hydrophilic cases were found to be favorable for recovering oil, despite greater interfacial tensions, due to wettability alteration towards water-wet conditions and the formation of low apparent-viscosity oil-in-water (O/W) macroemulsions. Wettability alteration and bubble-oil pinch-off were identified as contributing mechanisms to the formation of these macroemulsions. Conversely, characteristically less hydrophilic cases were accompanied by a large increase in apparent viscosity due to the formation of water-in-oil (W/O) macroemulsions.

## Introduction

Recent works have predicted an increase in the role of heavy oil in the global energy landscape^1–5^. This oil, with viscosity ~ 10–10^6^ cP, is typically produced by enhanced oil recovery (EOR) methods^2^. These methods involve injection of fluids into an oil reservoir to decrease the rate of decline in oil production^6,7^. One goal of EOR is to decrease the mobility ratio:

1 $$ M = \frac{{\lambda_{D} }}{{\lambda_{d} }} = \frac{{{\raise0.7ex\hbox{${k_{r, D} }$} \!\mathord{\left/ {\vphantom {{k_{r, D} } {\mu_{D} }}}\right.\kern-\nulldelimiterspace} \!\lower0.7ex\hbox{${\mu_{D} }$}}}}{{{\raise0.7ex\hbox{${k_{r, d} }$} \!\mathord{\left/ {\vphantom {{k_{r, d} } {\mu_{d} }}}\right.\kern-\nulldelimiterspace} \!\lower0.7ex\hbox{${\mu_{d} }$}}}} $$

where $\lambda$ is mobility, ${k}_{r}$ is relative permeability, and $\mu$ is viscosity. The subscripts denote the displacing phase $D$ and the displaced phase $d$. Therefore, decreasing the viscosity of the heavy oil phase lowers $\lambda$ which leads to greater oil recovery. To this end, thermal EOR methods have historically been the most successful at achieving viscosity reduction and recovery of heavy oil^7^. On the other hand, the efficiency of these methods depends largely on the reservoir properties. For example, in deep formations at high pressures, higher temperatures are necessary to generate steam^7^. Thus, those cases could require prohibitively large energy consumption or CO_2_ generation. These drawbacks motivate investigation of alternative EOR processes for recovering heavy oil.

**Figure 1:**
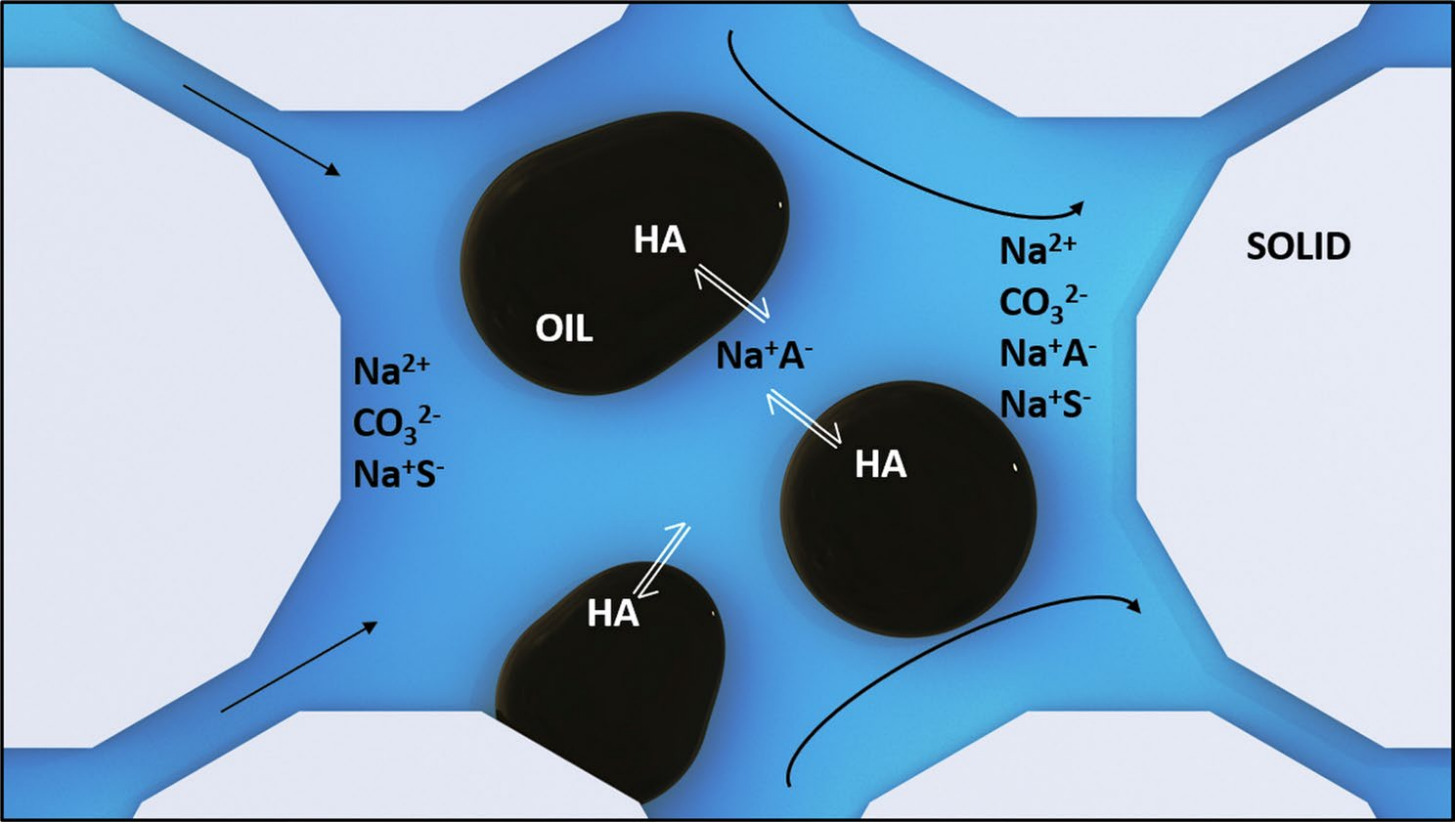
A schematic of alkali-surfactant EOR based on that of deZabala and Radke^36^. HA = Acidic components of the crude oil. Na^+^A^−^ = neutralized Acids or soaps. Na^+^S^−^ = synthetic surfactant component of injection solution.

Alkali Surfactant (AS) flooding is one such process that has been effective for recovering heavy oil at the lab scale^4,8–14^. AS floods involve an alkali component such as NaOH, sodium orthosilicate, sodium borate, or sodium carbonate that reacts with the naphthenic acid components present in the crude oil to produce soaps (see Fig. 1)^15^. A synthetic surfactant component is also present in this type of chemical flood. The primary mechanisms of action for AS floods are wettability alteration, interfacial tension (IFT) reduction, and emulsification^16^. Among these phenomena, the formation of macroemulsions, a process that depends on both wettability and IFT, stands out as a promising method for mobility control.

An emulsion is a colloidal dispersion of a discontinuous liquid phase existing as tiny droplets in an immiscible continuous liquid phase. In these dispersions, the viscosity is more like that of the continuous phase. Therefore, oil-in-water (O/W) emulsions could have a viscosity closer to that of the water than that of the oil^17^. In porous media, provided the oil saturation is below a certain value^18^, emulsifying the oil as an oil-in-water emulsion can similarly decrease apparent viscosities. Several researchers have demonstrated the promising process of recovering heavy oil as a low viscosity oil-in-water emulsion^13,19–22^.

Despite the success of these studies, poor mobility control (i.e. an unfavorable mobility ratio) has been identified as a prominent shortcoming of AS flooding for heavy oil recovery^15^. Some researchers have begun investigating methods to improve mobility control with AS EOR. Foam and polymer-foam EOR methods have been of interest due to the rheological properties of foam that promote mobility control ^23,24^. The “smart rheology” of foam results in improved mobility control despite extreme permeability and/or viscosity ratios^25–33^. When AS and foam are combined, the process is coined alkali-surfactant-foam (ASF) flooding. Gou et al. demonstrated this process can recover up to 94% of conventional oil from sandstone cores^34^. Telmadarrie and Trivedi showed that polymer enhanced foam could recover up to 70% of the oil in a fractured micromodel^24^. Also, Farzaneh and Sohrabi demonstrated an ASF process that could recover more than 90% of a heavy oil from a micromodel^1^. While these works demonstrate promise for ASF flooding, a proper application of phase behavior analysis is needed to achieve favorable oil-aqueous physiochemical interactions^35^.

Phase behavior of oil–water-surfactant systems were thoroughly described by Winsor^37,38^, and Healy et al. related Winsor’s methodology to microemulsion flooding^39^. These emulsion systems are classified by length scale. Microemulsion droplets are generally tens of nanometers in diameter, whereas those of macroemulsions are typically microns to millimeters in size. Focusing on microemulsions, Winsor defined the following: Winsor I (WI) systems are those in which the surfactant is more energetically stable in the aqueous phase exhibiting a thermodynamically stable lower-phase oil-in-water (O/W) microemulsion. Winsor III (WIII) systems are those in which the surfactant is energetically stable in a bicontinuous microemulsion middle layer in coexistence with bulk oil and water phases. Lastly, Winsor II (WII) systems are those in which the surfactant is more energetically stable in the oil phase exhibiting a thermodynamically stable water-in-oil (W/O) microemulsion. Surfactants systems exhibiting classical phase behavior can pass monotonically from WI to WIII to WII when changing a single system variable, such as salinity, temperature, oil type, and surfactant molecular weight, which alters the hydrophilic-lipophilic-balance (HLB). While microemulsions are thermodynamically stable fluids, macroemulsions are kinetically stable. Systems in which microemulsions are present will also tend to form macroemulsions of the same type (e.g. O/W or W/O) when perturbed by the shearing and capillary forces of flow in porous media.

Interpreting phase behavior of complex AS/crude oil systems can be obscured by phenomena such as the formation of multiple phase layers, partitioning of different surfactant species in the molecular weight distribution, and kinetically stable macroemulsions. The type of surfactant(s), brine, and alkali that are selected, and the chemical composition of the crude oil to be recovered, will all influence the resulting phase behavior and, consequently, the type(s) of macroemulsions and microemulsion phases that are likely to form during the ASF process. Researchers are examining the influence of phase behavior on heavy oil recovery^21,40^, but further work is necessary to optimize the process.

In this work, the ASF EOR method is demonstrated with a blend of two surfactants. A novel phase-viscosity plot or “map” of salinity versus soap fraction is first described. The map is a convenient representation of phase behavior and apparent emulsion viscosity data over a range of pertinent variables for the ASF process. Discussed next are micromodel floods that were conducted at characteristic soap fractions and added salinities corresponding to locations on the phase-behavior viscosity map. Oil recoveries and apparent viscosities were measured during the floods. It was found that the most hydrophilic system recovered the most oil at the lowest apparent viscosity. Conversely, the more hydrophobic systems recovered less oil at a much higher apparent viscosity. Several pore-scale observations are made to describe key differences among the flow tests.

## Materials and methods

### Chemicals

The crude oil selected for this study is a typical heavy crude oil from Kuwait. The oil was first centrifuged at 3,000 rpm and 30 °C for 9 h to separate any solids and water that may have been present. The oil was then decanted into a collection vessel and subsequently pumped through a 0.5 µm filter to further remove fines. The oil viscosity is 5,855 $\pm$ 40 cP at 22 °C and the total acid number (TAN) is 1.2 mg KOH/g oil. The viscosity was measured using a rheometer with a couette geometry. The TAN was measured by non-aqueous titration^41^. The Na_2_CO_3_ and NaCl were purchased from Sigma-Aldrich and used as received. The surfactants selected for this study were internal olefin sulfonate 15–18 (IOS) (ENORDET O320, Shell) and lauryl betaine (LB) (Solvay). The surfactant solubility testing and surfactant-blend screening process are discussed in the Supplementary Information text S1. IOS is a commonly tested surfactant in chemical EOR studies and LB has been demonstrated to be a foam booster in the presence of oil^42–45^. The molecular weights of IOS and LB were estimated to be 350 g/mol and 271.4 g/mol respectively.

### Phase behavior and falling sphere viscosity measurement

The Na_2_CO_3_ concentration, fixed at 0.5 wt%, was in stoichiometric excess such that all the acidic components in the oil could potentially react to form soaps. The surfactant ratio was fixed at IOS:LB 1:1, and the surfactant concentration was varied by diluting 5 wt% concentrated surfactant stock solutions with brine. The added salinity from NaCl—meaning salinity in addition to that from the alkali salt—varied from 0.5 to 6.0 wt%. These conditions are summarized in Table 1.

**Table 1:** Phase behavior conditions: pipette contents summary.

Injection component concentration(s) (wt%)		
Na_2_CO_3_	NaCl	IOS:LB 1:1
0.5	0.5, 1.0, 1.5, 3.0, 6.0	0.0, 0.02, 0.05, 0.1, 0.2, 0.3, 0.5, 1.0

The phase behavior systems were contained in 5 ml borosilicate glass pipettes that were flame-sealed using an acetylene-O_2_ torch. First, the tips of the pipettes were sealed, and then the tops were sealed after filling. The water–oil ratio (WOR) was fixed at 5:1. The fluids in sealed pipette were continuously rotary-mixed at a rate of 1 inversion every 30 s for 48 h and allowed to equilibrate for more than two weeks prior to phase classification. Because the heavy crude oil was less dense than the aqueous phases, conventional phase behavior was observed with the oil-rich phase floating on top of the water-rich phase. All systems were prepared and analyzed at ambient conditions.

Information about the phase behavior and the apparent viscosity for a given crude oil/surfactant/brine system at equilibrium is organized into a phase-behavior viscosity map. The phase-behavior portion of the map was constructed based on the Winsor classifications by visual observation of the samples two-weeks post-mixing. One indication that chemical equilibration was reached after two weeks was the agreement with trends of previous work^39^. After the samples physically equilibrated for 410 days, the apparent viscosity of the upper-oleic layer was measured in situ via falling sphere viscometry^46^. (See Supplementary Text S2 for more details about equilibration.) The viscosity was first measured for the undisturbed samples. Then the samples were mixed by ten inversions of the fluid layers, and the apparent viscosity measured again. The viscosity portion of the map was based on the latter measurement.

The phase-behavior viscosity map is plotted as the log of added salinity (wt% NaCl) vs. the soap fraction (Eq. 2)^47^.

2 $$ X_{soap} = \frac{{C_{soap} }}{{C_{soap} + C_{surfactant} }} $$

where ${X}_{soap}$ is soap fraction, ${C}_{soap}$ is the total concentration of soap generated by the alkali-naphthenic acid reaction in the sample, and ${C}_{surfactant}$ is the total concentration of synthetic surfactant in the sample pipette. The soap concentration is determined from the TAN assuming an excess of Na_2_CO_3_. By plotting salinity vs. soap fraction, the phase behavior can be described for different WOR^48^.

### Micromodel fabrication and experimental setup

**Figure 2:**
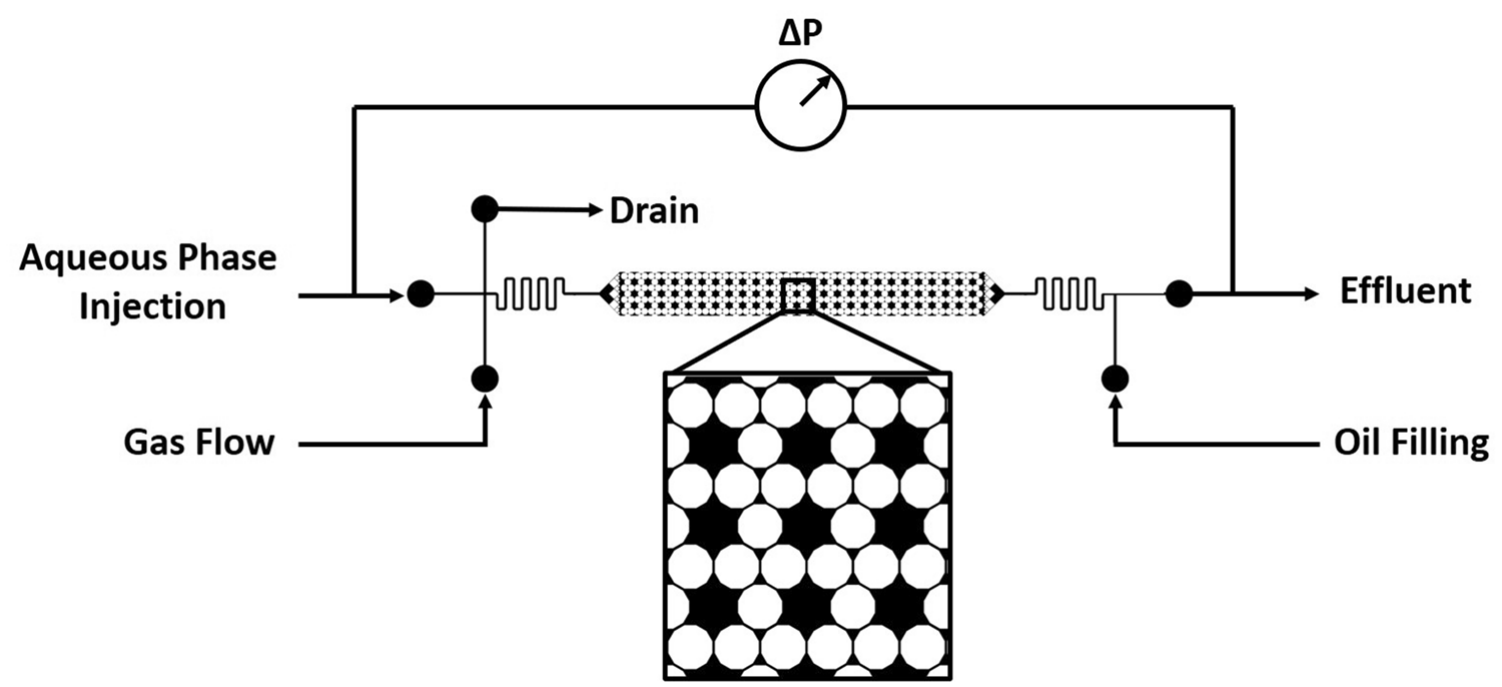
Experimental flow schematic. The inset is a detailed view of the micromodel pattern where white = solid and black = pore space.

The flow experiments were conducted in micromodels. The micromodels are constructed from NOA 81 polymer (Norland) following a soft-lithography protocol as previously reported in the literature^49,50^. This platform was selected because the pore-scale phenomenon of the flow process may be directly observed via a light microscope while simultaneously obtaining pressure-drop data. The properties of the micromodel are summarized in Table 2. The micromodel pattern and flow schematic are illustrated in Fig. 2. The intrinsic water-advancing contact angle of NOA 81 polymer is approximately 70° in air. Wettability alteration behavior by surfactants has been observed for this material^49^.

**Table 2:** Micromodel properties.

Micromodel property	Value
Channel dimensions (L × W × H)	2.6 cm × 0.32 cm × 35 µm
Grain diameter	450 μm
Pore throat size	35 μm
Pore throat : pore body ratio	15:1 or 3:1
Porosity	37%
Permeability	2.4 Darcy
Pore volume	1.1 μl

For all flow experiments, a microscope (Olympus SZX12) was used to view the micromodel at the pore-scale. The visual data was captured on a C-mount camera (IMPERX) and the pressure data was recorded with a 0 ± 50 psi pressure transducer (Valedyne). The liquid flow rates are controlled via syringe pump (KD Scientific). The gas flow rate is controlled using a calibrated 10 m glass capillary tube (SGE Analytical Science) with an internal diameter of 5 μm. This method allows for the delivery of gas at a constant flow rate regardless of down-stream pressure fluctuations^49^.

### Micromodel flow experiments

The micromodel was initially saturated via syringe pump with 6% NaCl brine and allowed to hydrate over-night. Heavy crude oil was pumped into the micromodel via the oil filling port. This step continued until the water content in the micromodel was reduced to connate water saturation (typically < 1%). Following this step, the micromodel surfaces were observed to be mixed-wet. Then, the micromodel was water flooded with 3 wt% NaCl brine. All steps were performed at ambient conditions. The flow rate and superficial velocity of the water flooding step were 4 µl/hr and 4 ft/day respectively. The capillary number is given by

3 $$ Ca_{w} = \frac{\mu u}{{\sigma^{ow} }} $$

where $\mu$ is brine viscosity, $u$ is superficial water velocity, and ${\sigma }^{ow}$ is oil–water IFT. The interfacial tensions in this study were measured using the pendant drop technique (KSV CAM-101) or the spinning drop technique (KRÜSS SITE 100). ${Ca}_{w}$ was 7.2 × 10^−8^ for all water floods. Aqueous blue food dye (2 wt%) was added to the brine for visual contrast with the solid grains. The micromodel was water flooded until residual oil saturation was reached. Alkali-surfactant solution was then injected into the micromodel at the same rate until the residual oil saturation was reached. Residual oil saturations for these first two steps were typically achieved after approximately 100 pore volumes of injection. Dry N_2_ gas was then co-injected with the alkali surfactant solution at a total superficial velocity of 4 ft/day. The foam was generated in situ in the porous media at a quality of 50% (where foam quality is the ratio of the gas volumetric flow rate to the sum of the gas and liquid volumetric flow rates). ASF flooding continued until residual oil saturation was reached. Residual oil saturation for this step was typically achieved after 10 pore volumes of injection.

In contrast to the equilibrated phase behavior tests, the unsteady dynamic nature of the flow experiments necessitates a simplification to make use of the information from the phase-behavior viscosity map. Thus, the characteristic soap fraction was chosen as the identifying abscissa value on the map for a given experimental condition. The characteristic soap fraction (Eq. 4) is calculated at the residual oil state after water flooding^51^:

4 $$ X_{soap}^{Sor} = \frac{{C_{soap } S_{orw} }}{{C_{soap } S_{orw} + C_{surfactant} \left( {1 - S_{orw} } \right)}} $$

where ${C}_{soap}$ is maximum total concentration of soap that can be generated by reaction with the alkali at residual oil saturation after water flooding. ${S}_{orw}$ is residual oil saturation after water flooding. ${C}_{surfactant}$ is total concentration of synthetic surfactant added to the brine.

Micromodel flow studies were carried out at several characteristic points on the phase-behavior viscosity map. The conditions for each experiment are summarized in Table 3. Note: Arabic numerals were utilized in the naming convention of the flow experiments (FE1-, FE1, FE3, and FE2) to distinguish these dynamic flow tests from the phase behavior (WI, WIII, WII).

**Table 3:** Summary of Micromodel Flow Experimental Conditions.

Experiment label	Characteristic soap fraction ${X}_{soap}^{Sor}$	Added salinity (wt%)	Corresponding IFT (mN/m)	Corresponding Winsor behavior ex situ
FE1-	0.19	1.0	4.49 ± 0.1	WI
FE1	0.54	0.5	3.64 ± 1.0	WI
FE3	0.55	3.0	0.15 ± 0.05	WI
FE2	0.94	3.0	3.6 ± 0.47	WII

Oil saturations were determined by image analysis. Micrographs of the entire porous media channel were taken and digitally stitched together to generate a high-resolution panorama. The time to take a single panorama was approximately 45 s or 0.03 pore volumes. These panoramas were analyzed using a custom MATLAB script that employed a local thresholding algorithm that normalized the stitched images. The apparent viscosity was calculated from rearranged Darcy’s law:

5 $$ \mu_{app} = - \frac{k}{u}\left| {\nabla p} \right| $$

where ${\mu }_{app}$ is apparent viscosity, $k$ is specific permeability, and $\left|\nabla p\right|$ is pressure gradient.

## Results and discussion

### Phase behavior

**Figure 3:**
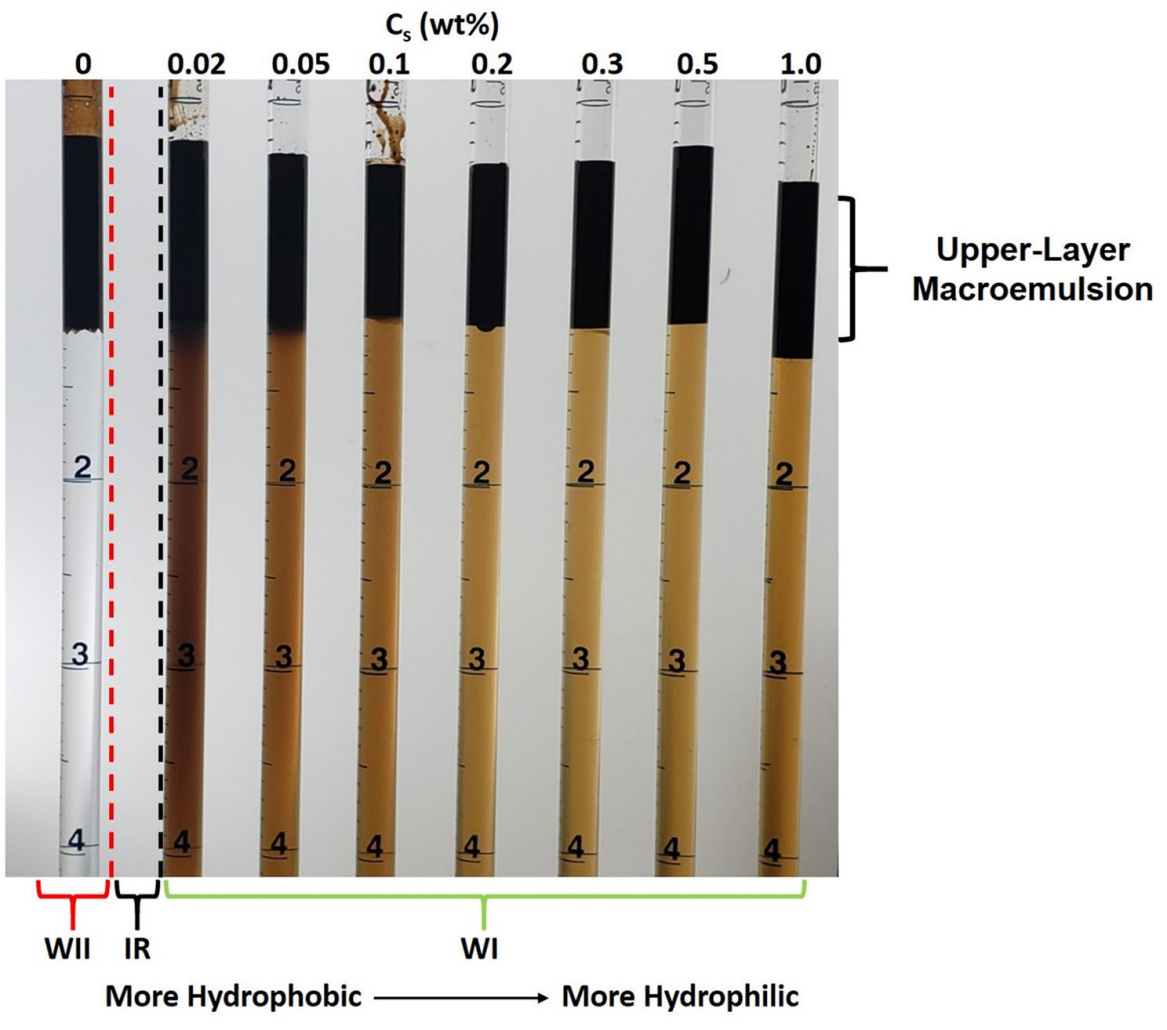
Example of the surfactant-concentration scan conducted at fixed 1.5 wt% NaCl and 0.5 wt% Na_2_CO_3_ after 410 days of equilibration. The Winsor behavior was unchanged from the two-week time point post-mixing. The surfactant/soap system becomes more hydrophilic as the concentration of synthetic surfactant in the system increases. In a more hydrophilic system, a greater fraction of the potentially surface-active components will be in the aqueous phase. A vertical red dashed line indicates the transition from WII systems, and a vertical black dashed line indicates the transition boundary to WI systems. The gap between these boundaries contains the inversion region (IR) in which WIII systems might be found.

Figure 3 is a photographic example of the surfactant-concentration scan conducted at 1.5 wt% NaCl and 0.5 wt% Na_2_CO_3_. In addition to the classical microemulsion phases, all samples exhibited an upper-oleic layer macroemulsion that was either water-in-oil or oil-in-water. In all systems, the macroemulsions that formed were of the same type as the microemulsion system. For example, systems containing WI microemulsions also exhibited oil-in-water macroemulsions. Also, the tendency of the glass tubes to be more oil wet increased with the hydrophobicity of the chemical system each contained.

### Phase-behavior viscosity map analysis

**Figure 4:**
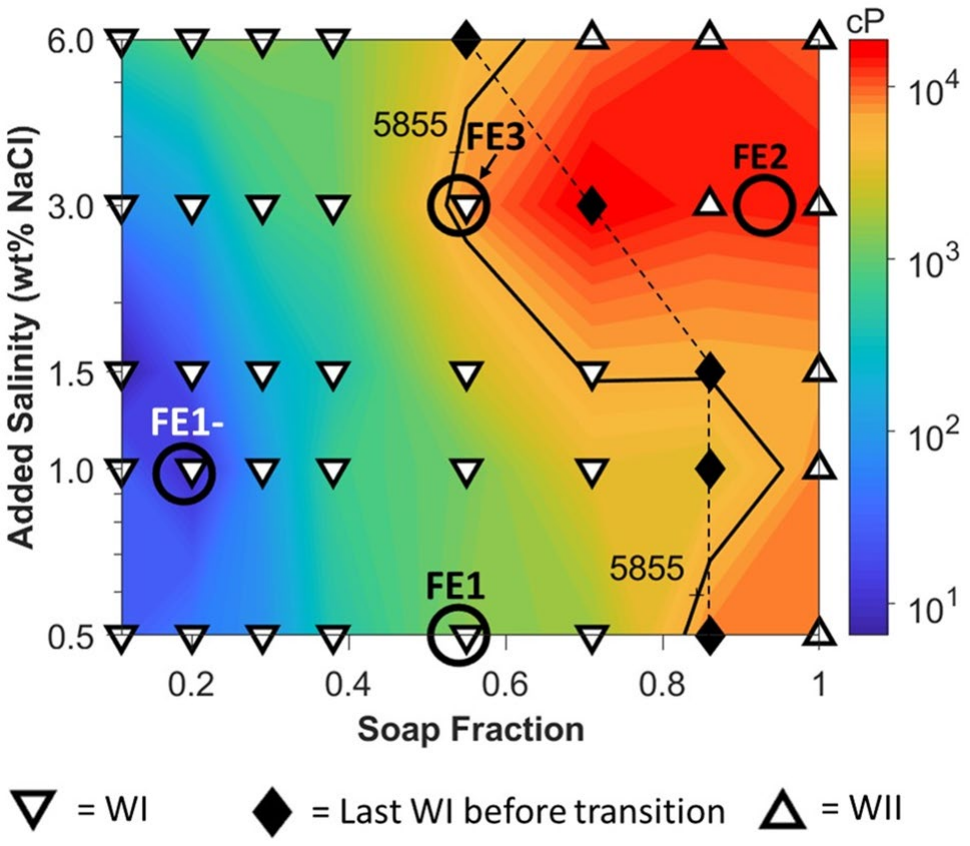
The phase-viscosity map (log of added salinity vs. soap fraction) constructed for a heavy crude oil in contact with a surfactant solution containing 0.5 wt% Na_2_CO_3_ and varying amounts of NaCl and LB/IOS. White triangles pointing downward (WI) and upward (WII) denote systems exhibiting lower- and upper-layer microemulsions respectively. Black diamonds represent WI compositions closest to the inversion region. These diamonds are connected via a black dashed line. The colors represent the log of apparent viscosity of the upper-oleic layer. The contour drawn in black represents the original bulk oil viscosity. The hollow black circles indicate the characteristic locations of flow experiments FE1-, FE1, FE3, and FE2.

From the phase-behavior viscosity map in Fig. 4, the trends in apparent viscosity of the upper-oleic layer with phase behavior may be readily distinguished by color. On this plot, the symbols represent the type of Winsor microemulsions system that was observed while the colors represent the magnitude of the measured apparent viscosity. The white triangles with the black borders and the black diamonds correspond to the phase behavior tests. White triangles pointing downward and upward denote systems exhibiting WI and WII microemulsions respectively. Black diamonds represent WI systems closest to the inversion region. Increasing the lipophilicity of the surfactant system results in the expected lower- to upper-phase microemulsion trend. This sequence occurs with increasing added salinity and increasing soap fraction (i.e. decreasing surfactant concentration). The colors of this plot represent the apparent viscosity of the upper-oleic layer on a log scale. The black contour is the bulk viscosity of the original heavy crude oil. The map in Fig. 4 was plotted based on the post-equilibration re-mixed apparent viscosity data. (See Supplementary Text S3 for a discussion on the effect of post-equilibration mixing on the phase-behavior viscosity map.)

A gradual increase in upper-layer apparent viscosity with increasing system hydrophobicity is observed for all lower-phase systems. This gradual viscosity increase may be the result of the macroemulsion type changing from single O/W to multiple W/O/W emulsions. These multiple emulsions should be more viscous than the single emulsions, but, because water remains the continuous phase, the viscosity is lower than that of W/O emulsions. The transition to upper-phase systems is marked by a significant increase in viscosity. This transition corresponds to the onset of W/O macroemulsions which are much more viscous than the bulk oil alone. For the particular synthetic surfactants of this study, these trends appear to be more sensitive to changes in soap fraction than salinity as evidenced by the relatively vertical orientation of the contour lines. These trends indicate that low-viscosity ASF flooding is most likely to be favorable in conditions near the bottom left of this map (i.e. low salinity and low soap fraction), and that increases in soap fraction should be more detrimental to the viscosity than increases in salinity.

### Micromodel flow experiment results

Micromodel flow studies were conducted at several characteristic points on the phase-behavior viscosity map. Point FE1- (soap fraction, salinity = 0.19, 1.0 wt%) is in the purple/blue region (10–100 cP) for apparent viscosity. Phase behavior near this point is well into the WI region and exhibits upper-layer O/W emulsions with apparent viscosities < 10 cP. This point was the most hydrophilic in nature with a relatively high surfactant concentration and low salinity. Point FE1 (0.54, 0.5 wt%), in the cyan/green region (100–1,000 cP), is the next most hydrophilic case. Phase behavior near point FE1 also exhibited upper-layer O/W emulsions and low apparent viscosity. By fixing soap fraction while increasing salinity, the orange region (5,000–10,000 cP) was obtained where point FE3 (0.54, 3 wt%) was selected. This case represents a system that is neither strongly hydrophilic nor strongly hydrophobic and exhibits a low IFT. Also, salinity was fixed but soap fraction was increased as denoted by point FE2 (3.0 wt%, 0.93) in the red region (> 10,000 cP). This case represents the most hydrophobic surfactant system among those tested.

#### Oil recovery

**Figure 5:**
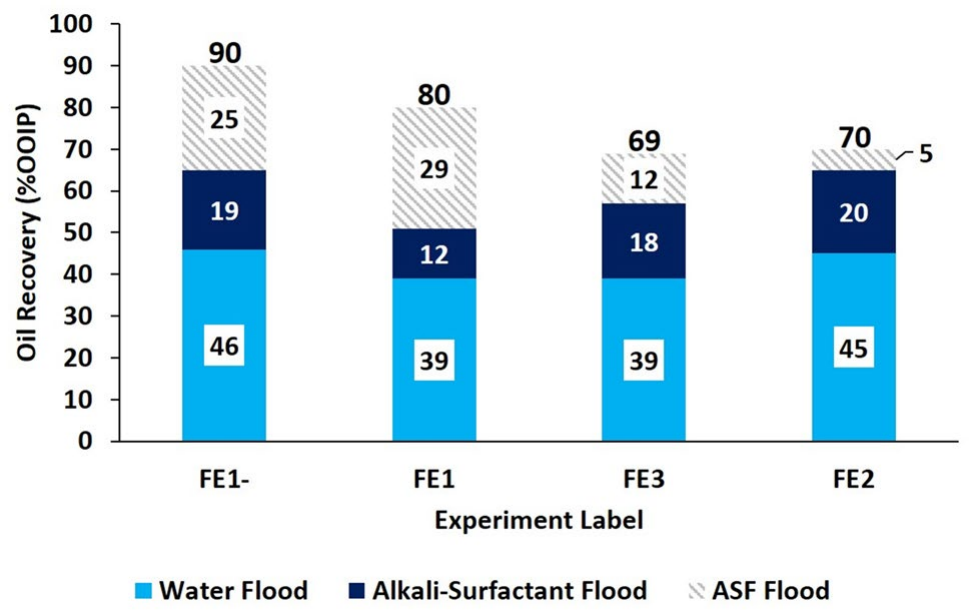
Oil recovery from micromodel flooding experiments. Light blue represents oil recovered by water flooding, dark blue represents oil recovered by the alkali-surfactant flood, and hashed grey represents oil recovered by alkali-surfactant-foam flooding. Each step was carried out until the residual oil saturation was reached. The total recovery for each flood is reported above the respective bar. In general, the more hydrophilic the flood, the more oil recovered.

The oil recovery from each flood is seen in Fig. 5. The FE1-flooding case resulted in approximately 10% more total oil recovery than that of experiment FE1 which in turn recovered 10% more total oil than that of experiments FE3 and FE2. The foam flood (ASF) was most effective for FE1 (29% improved oil recovery), and least effective for the FE2 flood (5% improved oil recovery). This implies that foam flooding provided less mobility control for more hydrophobic conditions during flooding.

#### Apparent viscosity and emulsion trends

**Figure 6:**
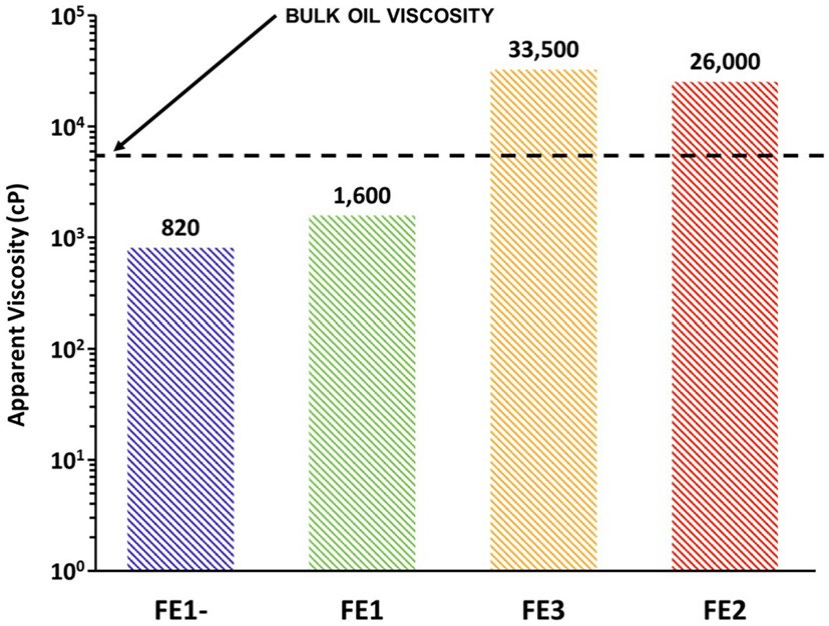
Average apparent viscosities during foam flooding. The hashed bars represent average apparent viscosity values calculated from pressure drop data recorded during the ASF flooding step of the micromodel experiments. The horizontal dashed line represents the bulk oil viscosity. Systems FE1- and FE1, containing O/W macroemulsions, exhibited characteristic apparent viscosities below that of the bulk oil. Due to the large oil recovery of 90% in system FE1-, and the multitude of injected pore volumes, the average characteristic apparent viscosity of 820 cP is most like that of the foam.

Because bulk emulsion viscosities can be significantly different from those in porous media, the magnitudes of apparent viscosities measured by falling sphere method should not be compared with those from flooding experiments. Therefore, only the general trends in both types of systems are discussed. The average apparent viscosities during foam flooding are reported in Fig. 6. The individual contributions of foam and oil to the apparent viscosity during ASF flooding are not distinguished; however, in the FE1- and FE1 cases, the total apparent viscosity was reduced below that of the original bulk oil. Conversely, floods FE2 and FE3 in the more hydrophobic regions of the phase-behavior viscosity map resulted in an apparent viscosity approximately 5 to 7 times greater than the bulk viscosity of the oil. These trends agree with what is implied by the phase behavior viscosity map of Fig. 4. Areas of the map exhibiting high apparent viscosities with tendency to form W/O emulsions correspond to large characteristic apparent viscosities in the micromodel experiments and vice versa for the low viscosity O/W bulk emulsion systems. Combing these tends with the knowledge of the oil recovered by ASF flooding from Fig. 5, implies that the mobility ratio in experiments FE1- and FE1 were more favorable than those of either FE3 or FE2.

**Figure 7:**
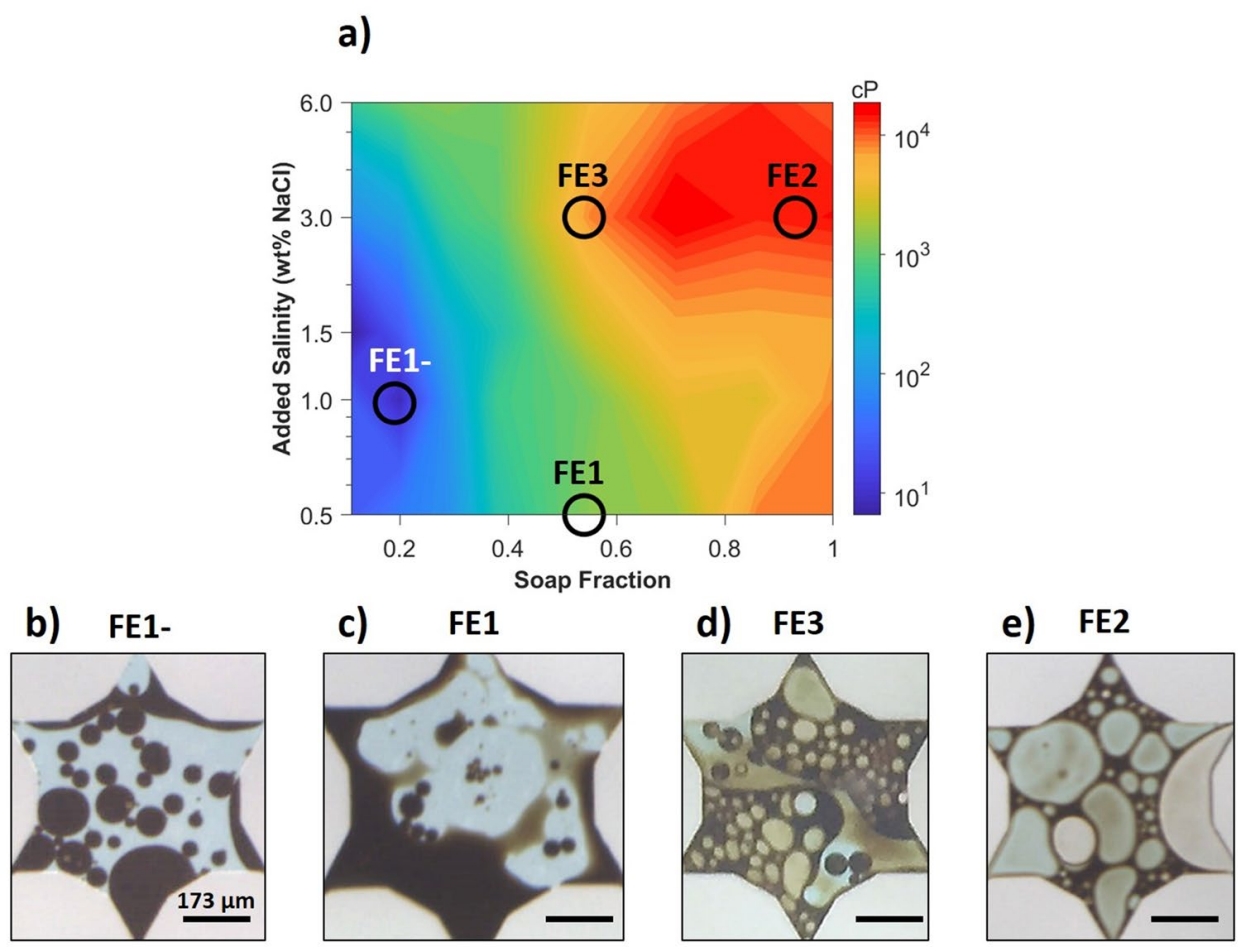
Typical single-pore fluid distribution for each flooding experiment. (**a**) A simplified phase-behavior viscosity map with the characteristic locations of each flood circled. (**b**) Example of a typical pore from the FE1- experiment undergoing AS flooding. An O/W emulsion is present and there is an absence of any oil film. (**c**) Example of a typical pore from the FE1 experiment undergoing AS flooding. O/W emulsion droplets are present, and a thin oil film convers some of the surfaces. (**d**) Example of a typical pore from the FE3 experiment undergoing AS flooding. O/W, W/O/W, and W/O emulsions are all present, and a thin oil film covers most surfaces. (**e**) Example of a typical pore from the FE2 experiment undergoing ASF flooding. A W/O emulsion is present, and a thin oil film covers all surfaces. All scale bars = 173 µm.

The typical progression in pore fluid distribution for each flooding case is visualized in Fig. 7. A representative image of a single pore was selected for experiments FE1-, FE1, FE2, and FE3. Figure 7a is a simplified version of the phase-behavior viscosity map in Fig. 4 with the phase behavior information removed to clearly indicate the selected conditions for the flow tests. The corresponding characteristic locations of each experiment are circled in black on the viscosity map. As seen in Fig. 7b,c, the low apparent viscosities of floods FE1- and FE1 are attributed to the formation of an oil-in-water macroemulsion. This emulsion type is favored, in part, due to the relatively more water-wet surfaces. Conversely, as seen in Fig. 7d,e, the water-in-oil emulsions that were predominately formed in cases FE2 and FE3 may explain the high apparent viscosities measured for those experiments. The wettability of these systems is more oil-wet, as evidenced by the presence of thin oil films, which favors the stabilization of W/O macroemulsions. In general, the phase viscosity map accurately supports the trends that were observed in the micromodel experiments, and this map could ultimately have utility for predicting the performance of other EOR processes involving phase behavior.

#### Pore-scale mechanisms

##### Water flooding

**Figure 8:**
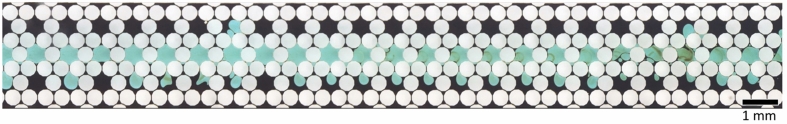
Representative image of micromodel state at the end of water flooding. Dark brown pores are oil-filled while blue/green pores are water filled. The light brown color in the water-filled pores indicates the presence of an oil film.

As expected, due to the unfavorable mobility ratio, water primarily fingered through a single pathway in the micromodel after water breakthrough. This pattern can be seen in a representative panorama of the middle half of the micromodel after water flooding (Fig. 8). Because all pore throats are the same, the remaining oil was unswept due to viscous instabilities alone. Following water flooding, the micromodel surfaces in all experiments were mixed-wet as evidenced by a light brown oil layer remaining in some of the water-swept pores. Due to the mixed wet nature of the surfaces, some water-in-oil macroemulsions formed by snap-off.

##### Alkali-surfactant flooding: wettability and emulsification

**Figure 9:**
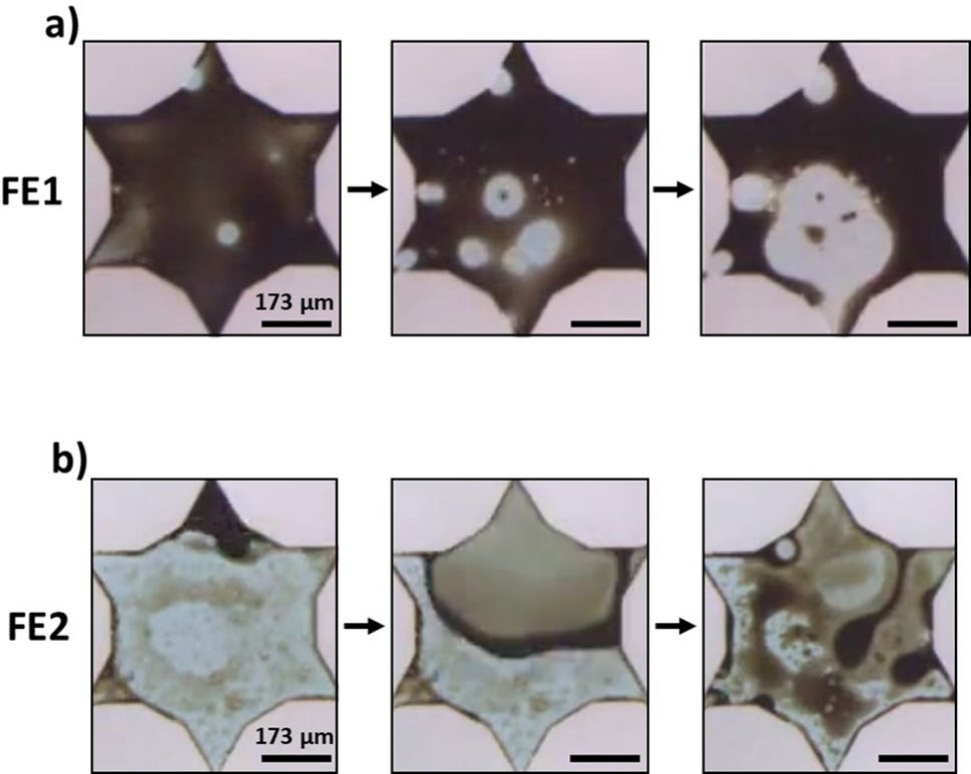
Wettability trends observed in the FE1 and FE2 case. The dark brown phase is the heavy crude oil, the light blue phase is the AS solution, and the light grey shapes are solid. (**a**) The more hydrophilic FE1 transitions toward a water-wet stated while (**b**) the opposite trend is exhibited in flooding case FE2. All scale bars = 173 µm.

Wettability alteration took place in the presence of alkali surfactant solution in all flooding cases. The wetted state of the micromodel was determined by the movement of oil films across the micromodel surfaces (Fig. 9). A receding oil film was interpreted as a transition toward a water-wet state while a spreading oil film indicated the surfaces were becoming more oil wet. In the FE1- and FE1 cases, the presence of the alkali surfactant solution resulted in receding oil films. In the FE2 and FE3 cases, the opposite trend was observed, and the surface became more oil wet.

**Figure 10:**
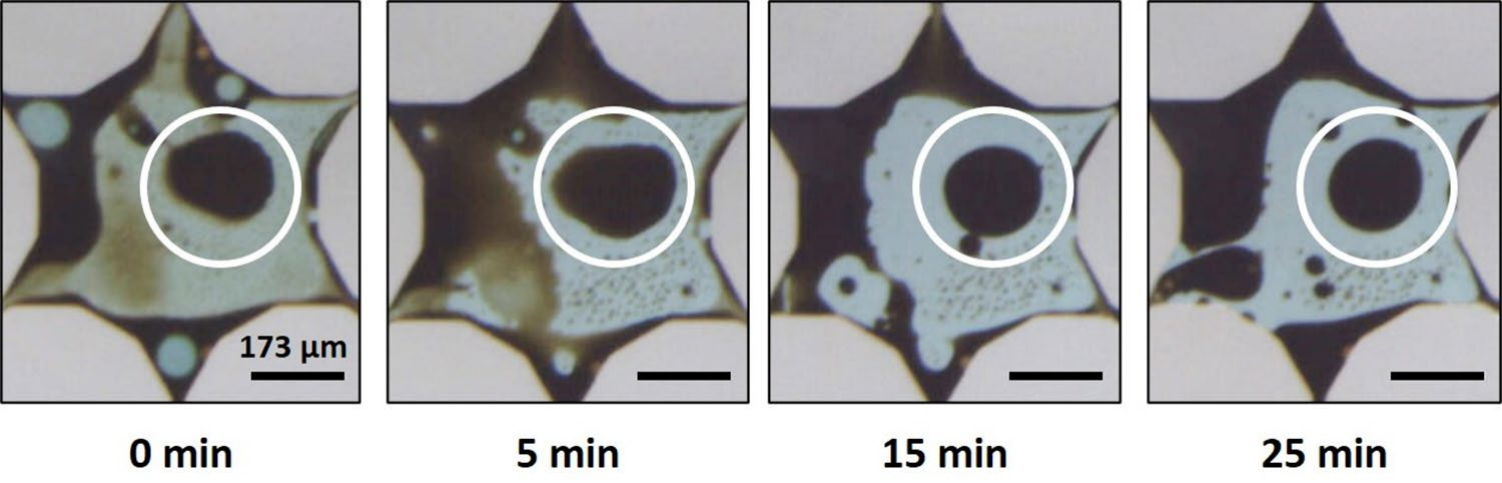
An example of macroemulsion formation by wettability alteration during the FE1- alkali-surfactant flood. An oil ganglion (circled in white) dewets from the surface of the micromodel over time to form an O/W emulsion droplet. All scale bars = 173 µm.

The types of emulsions that formed in each case corresponded with the characteristic apparent viscosities. W/O macroemulsions resulted in high values of measured apparent viscosity while O/W macroemulsions led to low values of apparent viscosity. Furthermore, the trends in wettability corresponded to the types of emulsions that formed during the flooding processes. Note: these observed trends should depend only on the wettability transition and are independent of the solid surface material. Flow channels with more water-wet surfaces resulted in generation of O/W macroemulsions while channels with more oil-wet surfaces formed W/O macroemulsions. In the FE3 case, multiple macroemulsions, tending to be W/O/W or water-filled vesicles, were observed in conjunction with W/O macroemulsions. An example of wettability alteration influencing the formation of macroemulsions is given in Fig. 10. In this case, for the FE1- experiment, O/W macroemulsions could form via de-wetting from the surfaces of the micromodel. Primarily, macroemulsions were formed by snap-off.

##### Alkali-surfactant-foam flooding

**Figure 11:**
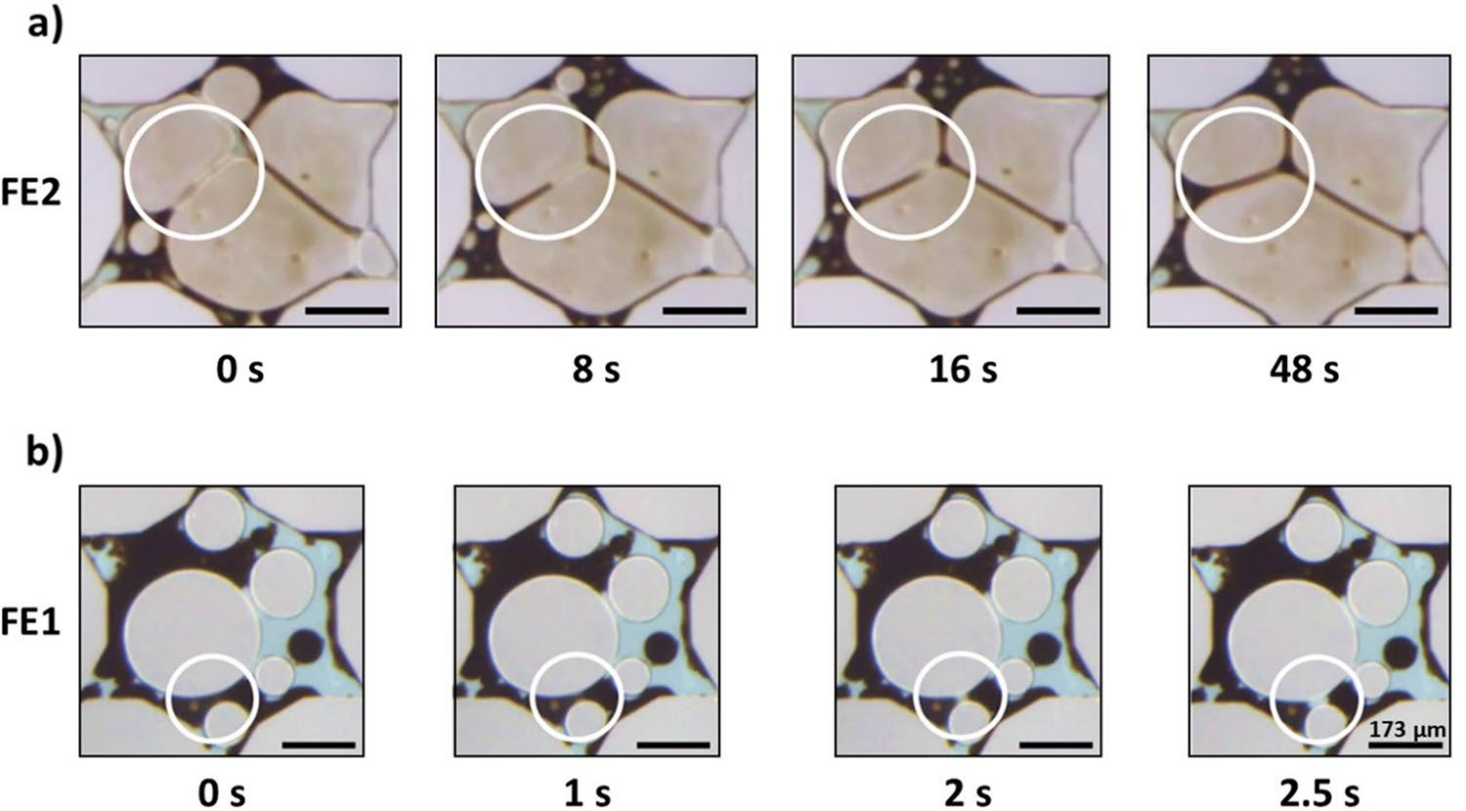
Snapshots taken from experiments during ASF flooding. (**a**) FE2 experiment: Oil spreads into lamellae, circled in white, but coalescence did not occur. (**b**) FE1 experiment: An oil O/W emulsion droplet, circled in white, is split in two by neighboring bubbles to form two smaller droplets. All scale bars = 173 µm.

Strong foam formed in situ in the porous media during co-injection. Slugs of gas broke up into smaller bubbles by pinch-off and lamella division. The primary foam-destruction mechanism for all cases was Ostwald ripening. The foam remained stable in the presence of the crude oil. As seen in Fig. 11a, for the FE2 case, oil tended to spread at the gas interfaces to fill lamellae, but the bubbles did not coalesce. In the FE1 and FE2 cases, a bubble-oil pinch-off mechanism occurred in which two bubbles were involved in splitting oil ganglia into smaller droplets (shown for case FE1 in Fig. 11b. This mechanism is similar to the bubble–bubble pinch-off described by Liontas et al.^52^, yet here all pinching entities are fluids similar to the phenomenon described by Vecchiolla et al.^53^.

## Conclusion

In this work, the impact of in situ emulsification of heavy oil by alkali surfactant foam enhanced oil recovery (ASF EOR) was explored. A novel phase-behavior viscosity map was proposed as an experimental method to aid in the rapid selection of optimal injection conditions for displacing heavy oil as a low-viscosity oil-in-water (O/W) emulsion. The map is constructed by arranging test results of phase behavior in sealed pipettes on a plot of the log of added salinity vs. soap fraction and by overlaying viscosities measured from the same test samples on said plot. The characteristic soap fraction ${X}_{soap}^{Sor}$ was selected as a benchmark to relate dynamic flow behavior in micromodel experiments to static phase behavior in sealed pipettes. The efficacy of ${X}_{soap}^{Sor}$ as a benchmark was demonstrated by evaluating four flooding experiments (FE1-, FE1, FE3, and FE2), at different characteristic soap fractions and salinities, for consistency with predictions informed by the phase-viscosity map. Foam was found to be stable across all flooding experiments.

In the most hydrophilic case (FE1-), 90% of the 5,855 cP heavy oil could be recovered at an apparent viscosity of 820 cP. This relatively favorable result was due to wettability alteration towards water-wet and the formation of low apparent-viscosity O/W macroemulsions. Conversely, the most hydrophobic case (FE2) resulted in a lower total oil recovery (70%) accompanied by a large increase in apparent viscosity, likely due to the formation of W/O macroemulsions, as predicted by referencing the phase-behavior viscosity map. Thus, the map method is shown to help rapidly identify promising injection compositions for recovering heavy oil via in situ O/W emulsification during ASF EOR. Additionally, wettability alteration and bubble-oil pinch-off were identified as contributing mechanisms to the formation of O/W macroemulsions in the more hydrophilic flooding experiments. Foam was more effective at recovering oil in these cases presumably due to more favorable mobility control.

## Supplementary information

Supplementary information
