# Supplementary material for: A systematic approach to alkaline-surfactant-foam flooding of heavy oil: microfluidic assessment with a novel phase-behavior viscosity map
Source: Sci Rep. 2020 Jul 31;10:12930. doi: 10.1038/s41598-020-69511-z (PMC7395747; doi:10.1038/s41598-020-69511-z)
Supplement: Supplementary file 1 — Supplementary information [file 41598_2020_69511_MOESM1_ESM.pdf]

Supplemental Information

A Systematic Approach to Alkaline-Surfactant-Foam Flooding of Heavy Oil: Microfluidic Assessment with a Novel Phase-Behavior Viscosity Map

Eric Vavra, Maura Puerto, Sibani L. Biswal*, George J. Hirasaki*

Rice University, 6100 Main St., MS-362, Department of Chemical and Biomolecular Engineering, Houston, TX, 77005 USA.

* To whom correspondence should be addressed:

email: biswal@rice.edu, gjh@rice.edu

Text S1: Surfactant Salinity/Blend Scan with IOS and LB

To select a blend ratio of IOS and LB, phase behavior salinity scan tests were performed. The salinity levels of 1.5, 3.0, and 6.0 wt% were selected to simulate low-salinity injection, sea water, and formation brine cases. The surfactant concentration, alkali concentration, and water-oil-ratio (WOR) were fixed at 1.0 wt%, 0.5 wt%, and 3:1 respectively. The surfactant concentration of 1.0 wt% was the maximum concentration tested in the construction of the phase-behavior viscosity map. The IOS/LB weight ratio was varied in 20 wt% increments from IOS only to LB only. The procedure was as follows:

1. 0.9 ml of 0.5 wt% Na2CO3 (Sigma-Aldrich) solution in DI water was transferred to a 15 ml glass vial.
2. A pre-determined amount of 10 wt% NaCl solution was then added to the vial.
3. A pre-determined amount of 5 wt% of each surfactant solution was then added to the vial.
4. The appropriate amount of DI water was added to bring the total aqueous volume to 9 ml.
5. 3 ml of the crude oil sample was added to the vial to bring the total volume to 12 ml. A WOR of 3:1 was used.
6. The vials were capped, mixed end-to-end overnight, and allowed to equilibrate for 10 days at room temperature.
7. Photos were taken of the equilibrated samples.

The contents of each vial are summarized in **Table S1**.

Table S1 Surfactant Salinity/Blend Scan for IOS/LB Crude Oil: Vial Contents Summary (WOR = 3:1)

**Total Surfactant Concentration (wt%)**	**Alkali (Na_2CO_3) Concentration (wt%)**	**Salt (NaCl) Concentrations (wt%)**	**Surfactant Blend Percentages (IOS/LB) (%)**
1.0	0.5	1.5, 3.0, 6.0	100/0, 80/20, 60/40, 40/60, 20/80, 0/100

The phase behavior results for the blend scan with the IOS/LB alkali system in contact with the crude oil is shown in **Figure S1**. A surfactant blend ration of IOS/LB 50/50 was selected because this ratio exhibited a WI to WII transition across the salinities of interest. The presence of this transition was necessary to examine the effect of Winsor type on flow behavior.

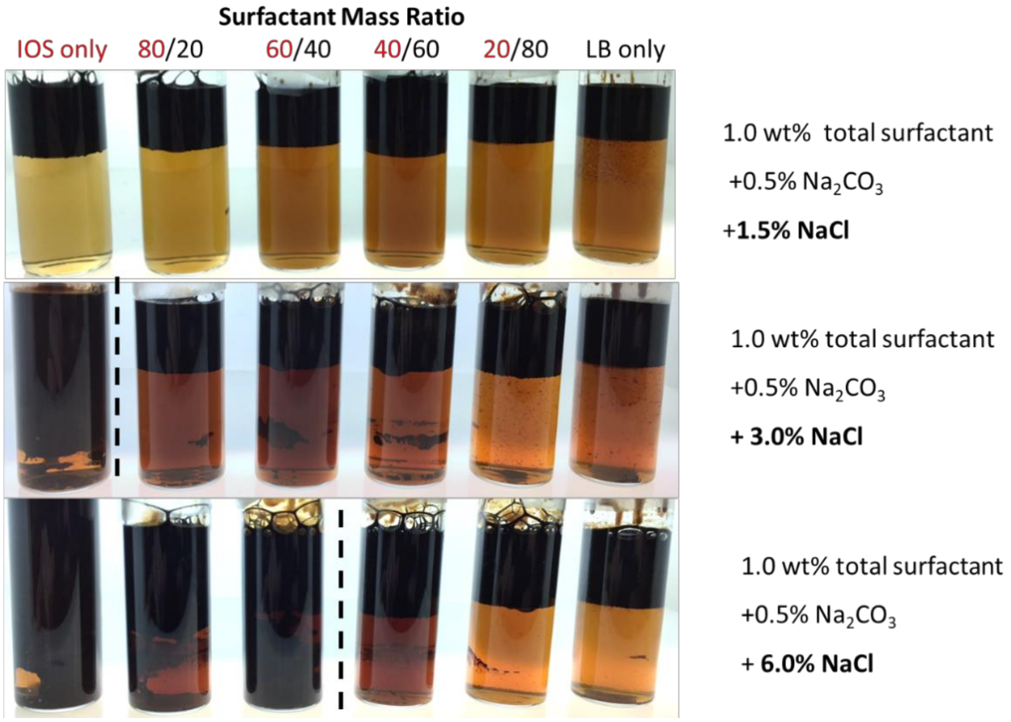

Figure S1. Phase behavior for surfactant salinity/blend scan. The vertical black lines indicate the transition from a Winsor I to a Winsor II system.

Text S2: Considerations for Chemical and Physical Equilibration

For the purposes of this study, equilibration of the alkali-surfactant phase behavior samples can be decoupled into two sets of processes: chemical and physical. Chemical equilibration processes take place on shorter timescales and are critical for Winsor classification. In this work, these types of processes include alkali reaction, surfactant diffusion, and micellization. Chemical equilibration is generally achieved with adequate mixing of the system and is complete when Winsor classifications no longer change with time. These classifications did not change from the two-week to the 410-day time points post-mixing and did not change for any sample after re-mixing. Physical equilibration, associated with longer timescales, affects viscosity measurements made by the falling sphere technique. These equilibration processes include phase settling and macroemulsion coalescence which can take years for systems with viscous oils that form stable macroemulsions. The 410-day time period was selected arbitrarily. During this time, our systems began to physically equilibrate; thus, the effects of physical equilibration on the falling sphere viscosity measurements could be determined as reported in the following section in this supplementary document.

Text S3: Effect of Post-Equilibration Mixing on the Phase-Behavior Viscosity Map

The effect of post-equilibration mixing on the viscosity measurements from falling sphere viscometer is interpreted from **Figure S2**.

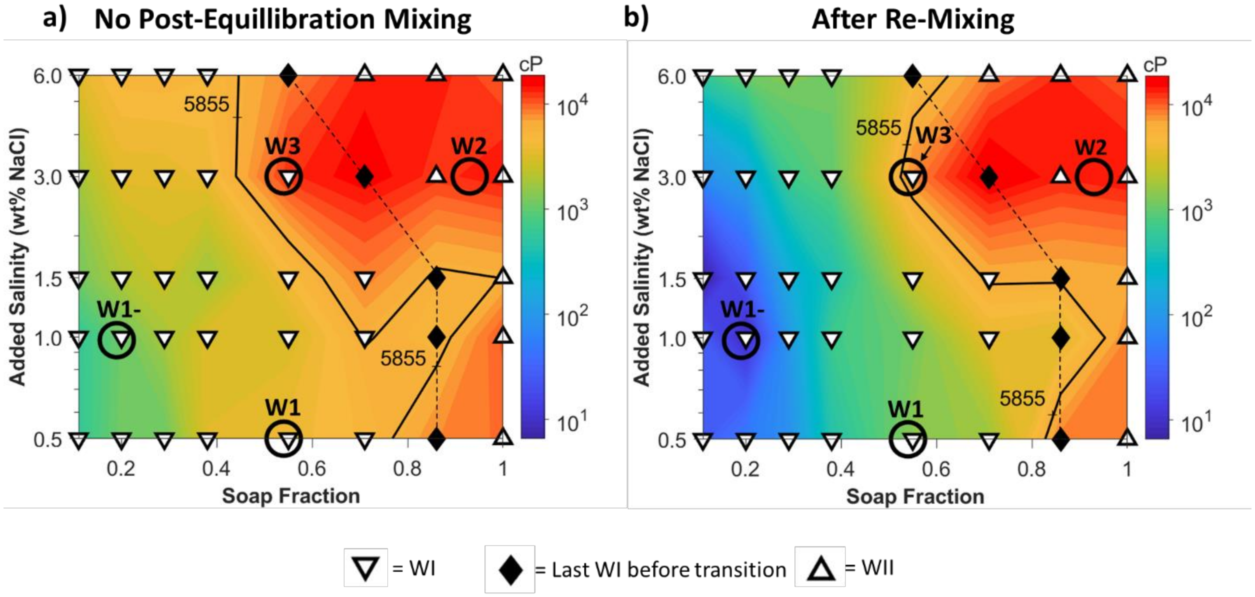

Figure S2. Effect of post-equilibration mixing on the phase-viscosity map. Map **a)** on the left is plotted with viscosity data measured after 410 days of equilibration before any re-mixing of the samples whereas map **b)** is plotted with data measured after the samples were re-mixed.

By comparing the color range on each plot, the effect of mixing before measuring the apparent viscosity becomes clear. The apparent viscosity values on the map with data for samples that remained undisturbed for 410 days, **Figure S2 a)**, spans from 600 to 16,500 cP while those measured immediately after mixing, **Figure S2 b)**, cover a wider and lower reaching range of 8.5 to 15,000 cP. Most likely, a thin layer of separated bulk oil was present on top of the upper layer macroemulsion in the aged undisturbed samples. This highly viscous oil layer contributed to the higher apparent viscosities of the WI samples compared to that of the post-mixed samples for which this layer would be emulsified into the macroemulsion layer. Between these two maps, the post-mixing data more closely captures the range of apparent viscosities observed from micromodel flooding. Thus, the plot in **Figure S2 b)** is better suited as a tool for prediction of the system behavior during dynamic micromodel floods.
